# Changes to the Intercondylar Ligaments of the Knee in Different Stages of Osteoarthritis—A Retrospective Cross-Sectional Study †

**DOI:** 10.3390/jcm14134513

**Published:** 2025-06-25

**Authors:** Elisabeth Mandler, Franz Kainberger, Lena Hirtler

**Affiliations:** 1 Center for Anatomy and Cell Biology, Medical University of Vienna, 1090 Vienna, Austria; elisabeth.mandler@meduniwien.ac.at; 2 Department of Biomedical Imaging and Image-Guided Therapy, Medical University of Vienna, 1090 Vienna, Austria; franz.kainberger@meduniwien.ac.at; 3 Teaching Center, Medical University of Vienna, 1090 Vienna, Austria

**Keywords:** knee joint, intercondylar notch, intercondylar space, osteoarthritis, anterior cruciate ligament, ACL, posterior cruciate ligament, PCL, anterior meniscofemoral ligament, posterior meniscofemoral ligament

## Abstract

**Background:** The intercondylar notch (IN) houses the central ligaments of the knee joint, namely the anterior and posterior cruciate ligaments (ACL and PCL) as well as the anterior and posterior meniscofemoral ligaments (aMFL and pMFL). As not only the available intercondylar space directly influences the encased ligaments, but also the ligaments themselves may influence each other, the purpose of this study was to evaluate the influence of osteoarthritis on central ligament morphology. **Methods:** Imaging data from the osteoarthritis initiative was used to assess 415 randomly selected patients, equally distributed across five groups based on osteoarthritis severity using the Kellgren and Lawrence classification. MRI scans were used to measure ligament structures in the coronal, axial and sagittal planes. The ACL was evaluated and classified into healthy, pathologic and ruptured. The relationship between osteoarthritis severity and the shape of the IN (A-shape, inverse-U-shape and Ω-shape) was analyzed in relation to ligament morphometrics and ACL condition. **Results:** The morphology of the ligaments is directly influenced by the development of osteoarthritis. In particular, the Ω-shape, which is associated with severe-grade osteoarthritis, is a risk factor for the development of ACL rupture (*p* < 0.001). But also, the condition of the ACL influenced the morphometrics of the posterior ligaments, and the PCL as well as the MFLs influenced each other. **Conclusions:** Statistically significant morphological changes to the encased ligaments in the intercondylar space in osteoarthritis were reported. In particular, the ACL shows a higher risk for pathological changes during ongoing joint degeneration due to osteoarthritis. The other evaluated ligaments—MFLs and PCL—are influenced by the condition of the osseous structures and the shape of the IN as well as by the condition and continuity of the ACL.

## 1. Introduction

A major part of the stabilizing structures of the knee joint is located centrally in the intercondylar notch (IN), specifically in the intercondylar space [1]. Here, the anterior (ACL) and posterior (PCL) cruciate ligaments connect femur and tibia, and the meniscofemoral ligaments (anterior—aMFL and posterior—pMFL) may run from the posterior horn of the lateral meniscus towards the femoral insertion of the PCL if present [2,3,4,5,6,7,8,9,10,11,12,13,14,15,16,17,18,19,20,21,22,23,24,25,26,27,28,29].

The morphology of the IN directly influences the structures within, as notch stenosis or the development of ridges and osteophytes may be a risk factor for ACL rupture and degeneration [30,31,32,33,34,35,36]. Especially as the ACL is one of the ligaments of the knee joint damaged most frequently, many studies focused on the pathophysiology of its rupture or degeneration. Impingement of the ligament on the lateral wall of the IN during knee movement, i.e., external rotation, flexion or abduction, is an important factor to consider as well as the corresponding principal stress on the non-impinged side and the stretching of the ACL over the PCL [36,37,38,39,40]. Different weight bearing conditions ranging from non-weight bearing to jump landing may additionally increase those stresses [38,41,42].

Compared with the studies available on ACL and its interaction with the IN, information on the interaction between the PCL or MFLs and the IN is scarce. In particular, the MFLs show a high variability [20,43,44,45], but have been shown to play a protective role for the posterior horn of the lateral meniscus [19,46,47,48] and to decrease the femorotibial contact pressure [49,50,51]. As they also inhibit posterior tibial translation together with the PCL and bear a part of the PCL’s load [26,50,52], they may play a major part in the outcome of injuries to the PCL, especially as they may stay intact if the PCL itself is ruptured [27,28]. Thus, the morphology of the MFLs and the PCL correlate with each other, and age has also been shown to be relevant, particularly influencing the morphology of the MFLs [53]. This is why the theory of a correlation between joint degeneration and especially the degeneration of the MFLs has emerged, although it has not yet been proven [21,24,54,55,56].

The aim of this study was therefore (1) to correlate the morphology of the IN in different grades of osteoarthritis with changes to the cruciate and meniscofemoral ligaments and (2) to define overall risk factors damaging the cruciate and meniscofemoral ligaments.

## 2. Materials and Methods

This study was conducted as a retrospective cross-sectional study, assessing the femoral condyles and intercondylar notch as well as the cruciate ligaments and meniscofemoral ligaments. Initial imaging data (n = 4796) was obtained through the osteoarthritis initiative (https://nda.nih.gov/oai, accessed on 1 August 2014). Ethical approval was granted by the responsible ethics board of the Medical University of Vienna (Nr. 1413/2014). All available knee radiographs were graded using the Kellgren and Lawrence classification [57,58].

To ensure balanced representation across groups, the number of individuals with the highest Kellgren and Lawrence grade (n = 83 patients) served as a reference. Participants for the remaining groups were then randomly selected and matched according to sex and side, following the protocol defined for the study groups in prior publications on the osseous morphology of the IN [59]: group 1 (grade 0), group 2 (grade 1), group 3 (grade 2), group 4 (grade 3) and group 5 (grade 4). As a randomization method, a stratified randomization was performed, in which the whole data set (n = 4796) was subdivided into subsets passed on the relevant covariates Kellgren and Lawrence grade, sex and sides. Within these subsets, cases were selected following a simple randomization by allocating random numbers. Based on these numbers, the first 83 patients were included.

A total of 415 patients were included, with a single MRI scan of one knee joint analyzed per individual.

### 2.1. Image Evaluation

Radiographic assessment and MRI measurements were conducted using available software tools (Osirix MD^®^ 12.0, Pixmeo SARL, Genève, Switzerland and Escape Medical Viewer^®^ 4.1.1, Escape OE, Thessaloniki, Greece).

Demographic data (see Table 1) and details of the specific morphology of the IN were obtained from a previous study [59]. The demographic data showed no significant differences between the groups concerning height, weight or BMI (*p*-values > 0.05). Patients without ortheoarthritis (Kellgren and Lawrence grade 0) were significantly younger (*p*-value < 0.001) than patients with ostroarthritis (Kellgren and Lawrence grades 1–4).

Coronal images from the study revealed that 52.8% (n = 219) of notches exhibited an Ω-shaped configuration, 43.4% (n = 180) an inverse-U-shape and 3.9% (n = 16) an A-shaped IN. In axial images, 41.7% (n = 173) of notches were Ω-shaped, 49.9% (n = 207) were inverse-U-shaped and 8.4% (n = 35) were A-shaped. These morphological variations correlated significantly with the severity of the osteoarthritis (*p* < 0.001). The inverse-U-shape was predominantly associated with early stages of OA (grade 0–1). A-shaped notches were less commonly observed and generally showed grade 0–2, while the Ω-shape was associated with more advanced OA (grade 2–4), typically characterized by the development of osteophytes contributing to narrowing of the IN.

**Figure 1:**
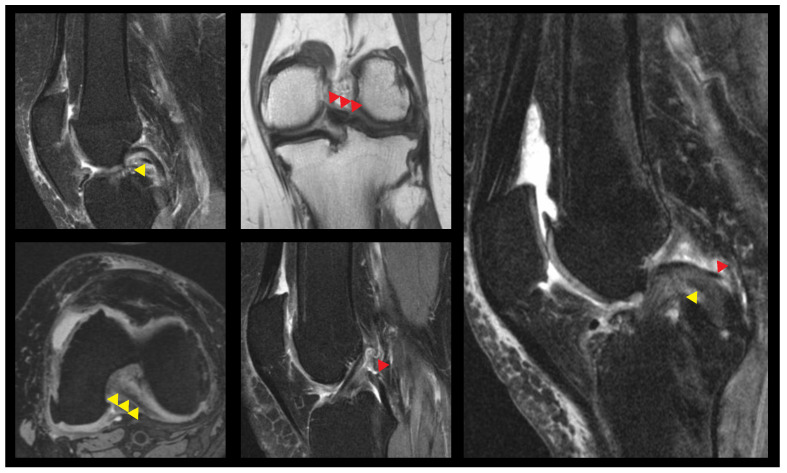
Exemplary cases of existing sole aMFL (yellow arrowheads), sole pMFL (red arrowheads) and both MFLs present (image on the right).

The following morphometrics of the cruciate ligaments were documented: cross-sectional area, sagittal and horizontal area in the axial plane, PCL-angle in the sagittal plane and morphology of the ACL (normal, total rupture, partial tear, cysts, edematous swelling). Concerning the meniscofemoral ligaments, their presence or absence was recorded (Figure 1). Measurements of length, anteroposterior diameter and cross-sectional area were obtained either in the axial or sagittal plane. Additionally, in the sagittal plane, the angulation of the pMFL was measured.

### 2.2. Statistics

Statistical analysis was performed using IBM SPSS 23.0 for Macintosh^®^ (Armonk, North Castle, Westchester County, NY, USA). Mean, median and standard deviation were calculated for all metric variables. Pearson’s correlation coefficient was used for normally distributed metric data, and Spearman’s rank correlation coefficient for non-normal distributions. Using Student’s *t*-test, differences between groups (gender and side) were tested for significance with normally distributed metric data. In non-normally distributed metric cases, the Mann–Whitney U test was used. A Chi-Square test was used for categorical variables. ANOVA was used for comparisons involving more than two groups, combined with Tukey’s (homogeneity of variance) or Games–Howell (non-homogeneity of variance) post hoc test. Significance was set at *p*-values of <0.05, and *p*-values <0.01 were considered highly significant. Bonferroni correction was applied in multiple testing.

To assess inter- and intraobserver reliability, measurements were repeated by the primary investigator and two additional examiners in a random sample of 100 patients. The intraclass correlation coefficient (ICC) was used to evaluate accuracy of measurement, with interpretation based on Landis and Koch [60]: 0.00–0.20 (slight), 0.21–0.40 (fair), 0.41–0.60 (moderate), 0.61–0.80 (substantial) and 0.81–1.00 (almost perfect) agreement.

## 3. Results

Information on the morphometry of the ligaments may be found in Table 2.

**Figure 2:**
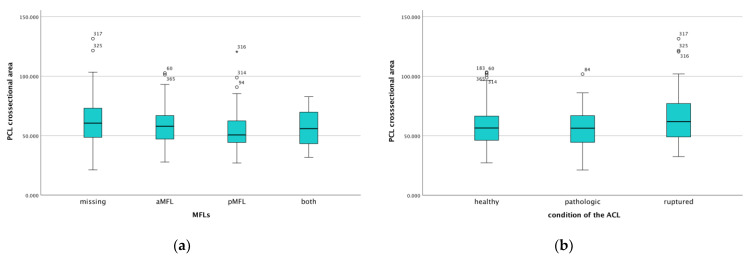
PCL cross-sectional area is influenced by (**a**) the presence/absence of the MFLs and (**b**) the condition of the ACL. Circles and asterisks indicate outliers with the specific case number.

The morphology of the MFLs was influenced by the presence or absence of either the aMFL or pMFL. In general, the cross-sectional area was significantly larger if one of the ligaments was absent compared to when both ligaments were present (*p* < 0.001). Additionally, the morphology of the PCL was affected by the presence of the pMFL. Specifically, when the pMFL was present, the cross-sectional area of the PCL was smaller than when the pMFL was absent (*p* < 0.001, Figure 2a).

The parameters of the PCL were significantly altered in the presence of a ruptured ACL compared to a healthy or pathologic but structurally intact ACL (*p* = 0.03, Figure 2b). In cases with ruptured ACL, the PCL exhibited a larger anteroposterior diameter, a smaller mediolateral diameter, an increased cross-sectional area and a reduced internal angle.

**Figure 3:**
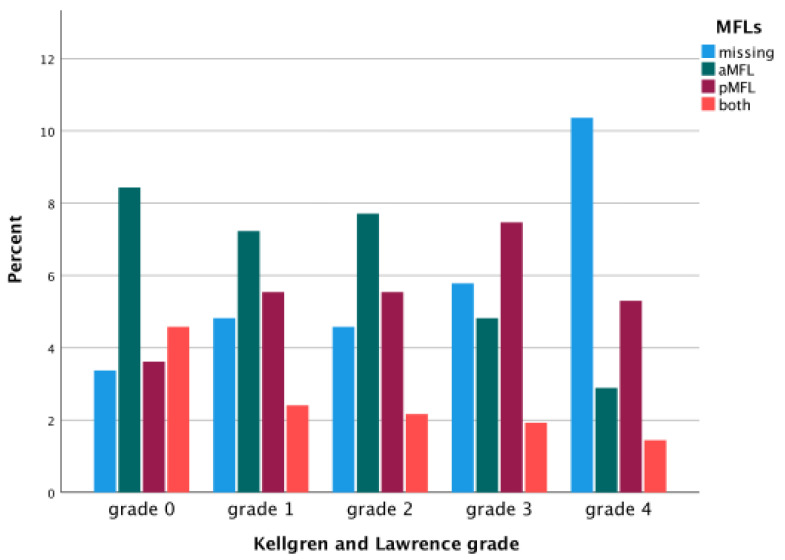
The presence or absence of the MFLs is influenced by the severity of osteoarthritis.

**Figure 4:**
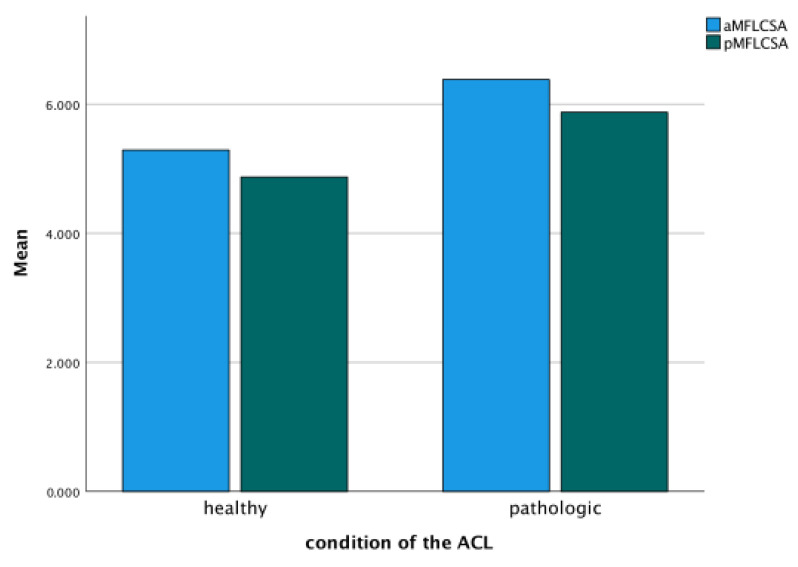
The cross-sectional area of the MFLs (aMFLCSA and pMFLCSA) is influenced by the condition of the ACL.

Furthermore, the severity of OA significantly influenced the measured values of the ACL (*p* = 0.00), the PCL (*p* = 0.00), and the aMFL (*p* = 0.00), but not of the pMFL (*p* = 0.53). The cross-sectional area of the ACL was significantly altered in OA grade 4 compared to all other grades (*p* = 0.41), whereas the cross-sectional area of the PCL was not significantly affected (*p* > 0.05). However, the presence of the MFLs was less frequent, the higher the Kellgren and Lawrence grade (Figure 3). Also, the condition of the ACL influenced the size of the MFLs (*p* = 0.00). The less healthy the ACL was, the larger the cross-sectional area of the aMFL and pMFL (Figure 4).

**Figure 5:**
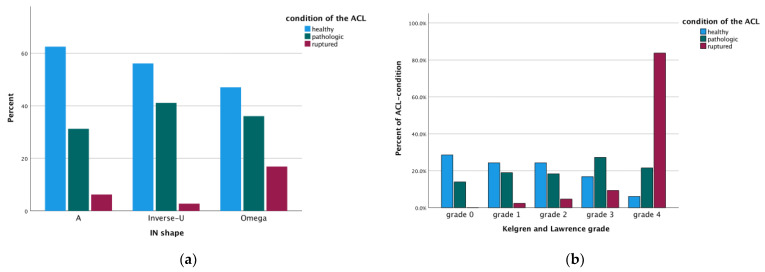
Correlation between ACL condition and associated anatomical and pathological factors: (**a**) there is a correlation between IN shape and condition of the ACL; (**b**) the more severe the osteoarthritis, the higher the risk for ACL rupture (*p* < 0.001).

**Figure 6:**
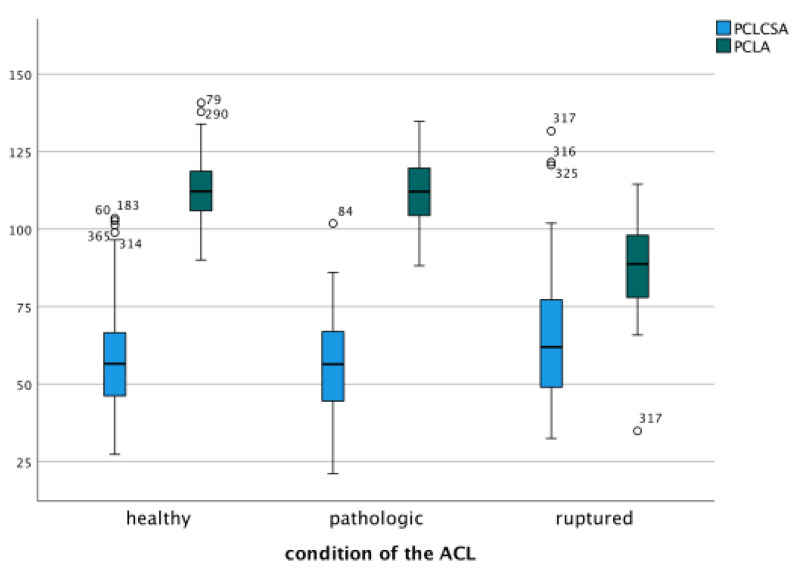
Parameters of the PCL such as cross-sectional area (PCLCSA) and intraligamentous angle (PCLA) were significantly different in cases with ruptured ACL compared to those with a healthy or pathological ACL. Circles indicate outliers with the specific case number.

Since the severity of the OA correlated with the shape of the notch, and the osseous morphology significantly influenced ligament morphology, a correlation was also observed between the shape of the IN and the ligaments (Figure 5a). A relationship was observed, with higher OA severity significantly associated with an increased likelihood of ACL rupture (*p* < 0.001). The condition of the ACL varied according to Kellgren and Lawrence grades, with grade 4 linked to complete ACL rupture (Figure 5b). Additionally, the parameters of the PCL were significantly different in cases with ruptured ACL compared to those with a healthy ACL or pathologic—but still continuous—ACL. Here, the cross-sectional area of the PCL was significantly larger and the intraligamentous angle of the PCL significantly smaller (Figure 6). The MFLs were generally not influenced by the shape of the IN (*p* < 0.001), except for the cross-sectional area of the pMFL, which was significantly larger in cases with an Ω-shaped IN than in cases with an inverse-U-shaped notch (*p* = 0.04).

Comparing the measurements of the cruciate ligaments, all values including the intraligamentous PCL-angle were significantly larger in males than in females (*p* < 0.13–0.001). In the MFLs, only the length of the ligaments was significantly larger in males than in females (*p* < 0.001); all other morphometrics showed no differences (*p* = 0.20–0.80).

### Differences Between Coronal and Axial Plane and Intra- and Interobserver Reliability

The intraobserver reliability showed a substantial to (almost) perfect repeatability in all ligamentous measurements (0.65–0.99). The interrater reliability showed a slight to (almost) perfect repeatability (0.2–0.91) in all ligamentous measurements.

## 4. Discussion

Results in this study clearly show a correlation between the severity of osteoarthritis and changes to the ligaments housed in the intercondylar space. These changes are mostly due to the alteration in shape of the IN towards an Ω-shaped notch, which showed the highest risk of ACL rupture.

The development of the Ω-shaped notch reflects, in part, the presence of central anteromedial and anterolateral ridges at the osteochondral border, which progressively develop into osteophytes as the severity of knee osteoarthritis increases [1,32,38,61]. These osseous changes are particularly prominent medially [62,63,64], and the more frequent alterations observed at the joint line closely reflect the overall pattern of progression seen in osteoarthritis. Through the development of central osteophytes, the available space, especially for ligamentous structures, diminishes within the intercondylar space. This subsequently results in impingement and eventually to rupture, particularly of the ACL in severe osteoarthritis [39,65,66,67,68,69,70,71,72,73,74,75,76,77,78,79,80,81,82]. More severe stages of osteoarthritis were shown to be a higher risk for ACL rupture [68], which this study confirmed (Figure 5b).

Rupture of the ACL initiates a vicious cycle that promotes further joint degeneration [83]. However, it is not only the ACL that is significantly affected by the development of OA; the PCL and aMFL also show correlated changes caused by osteoarthritis (*p* < 0.05), whereas the pMFL does not.

The fact, that the morphology of the IN influences ACL rupture risk has already been shown frequently in the current literature [31,32,37,39,69,70,72,74,84,85,86,87,88,89,90,91,92,93,94,95,96,97,98,99]. The data presented in this study showed a healthy ACL in 51.6% and a pathological or ruptured ACL in 48.4% of a cohort of 415 patients with varying grades of OA. The underlying pathologies severely influenced the morphometrics of the ACL.

The other three ligaments work synergistically to support knee joint function [13,26,27,50,52,100,101], yet they are not so prominently investigated in the literature as the ACL. However, they are also influenced not only by the dimensions or absence of each other (considering the MFLs), but also directly in their morphology by the shape of the IN and the condition of the ACL.

Although incidence rates of the aMFL and pMFL have been investigated in several prior studies, reported numbers show a high variability. In this study, the presence of at least one MFL (either pMFL or aMFL—58.5%) was overall lower compared to findings from other authors, who reported a range of 70.7–100%. The presence of the aMFL (43.6%) was similar to results of comparable studies in the literature, situated approximately in the middle of the reported range of 0–88.2% [19,20,21,22,29,47,56,100,102,103,104,105,106]. In this, the intimate relationship of the aMFL and the PCL may possibly be a reason for the rather large variance in findings, as the ligament itself is not always easily distinguishable, especially when using imaging techniques. The presence of the pMFL was reported to be only 40.0% in this study, which contrasts with findings from some authors, who observed the pMFL in nearly every case [29,103,104]. This positions the results of this study at the lower end of the range reported in the literature, which spans from 14.7 to 100% [19,20,21,22,29,47,56,100,102,103,104,105,106]. Here, the difference is likely due in part to the methodology. The majority of these articles describing a higher incidence of pMFLs were performed in anatomical specimens, which allows a closer and more detailed view of all ligaments involved during dissection. Possibly, the evaluation of MFLs using MRI leads to smaller ones being overlooked, especially since the reported incidence of MFLs is consistently lower in MRI than in anatomical studies.

One previously mentioned hypothesis addressing the different numbers of MFLs suggests that these ligaments degenerate during life, stating that patients with at least one MFL missing were likely to be of higher age than those with both MFLs present [25,56]. This theory is also supported by the data presented in this study, showing that the severity of osteoarthritis directly influences the presence of the MFLs (Figure 3) and is therefore tightly connected to the condition of the ACL (Figure 5). Here, especially the aMFL seems to be more acutely influenced by the severity of osteoarthritis, gradually showing lower numbers the more severe the underlying osteoarthritis was graded.

If only one MFL was identified, it was, on average, significantly larger than the corresponding MFL in knees where both MFLs were present (*p* < 0.001), as also reported by Roerich et al. [53]. This contradicts publications that attribute distinct functions to the MFLs, such as supporting the anterolateral or posteromedial bundle of the PCL, respectively [101]. However, it aligns with the findings of Cross et al. [107], who questioned the theory of the aMFL merely mimicking a PCL bundle. The present study suggests the hypothesis of an underlying developmental mechanism, through which a missing MFL can be compensated through the other present MFL, but further biomechanical studies will be needed, before there can be a final conclusion on this topic. This hypothesis would also be supported by looking at the embryological development of the MFLs [43], as the MFLs embryologically develop as a singular structure from the posterior horn of the lateral meniscus prior to the development of the PCL. Only through the later development of the PCL, which can split the initial single band into two depending on its position, are one (either aMFL or pMFL) or two MFLs (aMFL and pMFL) formed. This would especially explain why the existence or absence of the respective MFL would influence the cross-sectional area of the other.

Already mentioned was the collaboration between the MFLs and the PCLs discussed in the literature [27,101,107]. This can only be partly supported by the results of this study, as the cross-sectional area of the PCL was significantly smaller in cases with a pMFL present than if it was absent. The aMFL or the presence of both MFLs had no such effect. The pMFL is commonly described in the literature to be the larger of the two MFLs [19,21,24,29]; this also was not confirmed by the present results, which showed the two ligaments to be nearly equal in size. However, it supposedly supports the posteromedial bundle of the PCL synergistically [101]. Since the loading capacity of the posteromedial bundle reaches that of the anterolateral bundle only partly [13], the PCL may have to compensate a missing pMFL by being thicker and therefore stronger, whereas in the case of an absent aMFL, the reserve capacity of the anterolateral bundle may be enough.

### Limitations

Some restrictions of this study have to be kept in mind when interpreting its results. Due to its design as a retrospective cross-sectional study, imaging parameters could not be influenced to better suit the underlying research question. Through a prospective design, specific protocols could be designed to enhance ligamentous measurements, thus making them more reliable. However, intra- and interrater evaluation showed good reproducibility of the data. Additionally, one has to point out that although the overall sample size may seem statistically satisfying, the size of the different groups is rather small due to group and gender distribution. Direct comparison of the data to specific lifelong changes is also problematic due to the cross-sectional design. Here, however, one has to mention that long-term investigations, especially over several decades, are quite complex and also very costly in addition to asking for a long-term multicenter prospective design.

## 5. Conclusions

Statistically significant morphological changes to the encased ligaments in the intercondylar space were reported. In particular, the ACL shows a higher risk for pathological changes during ongoing joint degeneration due to osteoarthritis. The other ligaments—MFLs and PCL—are dually influenced by the condition of the osseous structures and the shape of the IN and by the condition and continuity of the ACL.

## Figures and Tables

**Table 1:** Demographic data of the study population [59] (reprint license: 6045820873878).

Variable	Average ± SD (Min–Max)
Age (years)	61.93 ± 9.22 (45–79)
Sex *	265 male (63.9%) 150 female (36.1%)
Side *	210 right (50.6%) 205 left (49.4%)
Race	295 (71.1%) Caucasian 108 (26.0%) black or African American 9 (2.2%) other non-white 3 (0.7%) Asian
Height (m)	1.72 ± 0.09 (1.48–1.90)
Weight (kg)	87.15 ± 16.57 (50.20–135.50)
BMI	29.47 ± 4.68 (19.50–44.60)

* distributed equally throughout the groups.

**Table 2:** Morphometrics of the ligaments. ACL = anterior cruciate ligament; PCL = posterior cruciate ligament; MFL = meniscofemoral ligament; aMFL = anterior meniscofemoral ligament; pMFL = posterior meniscofemoral ligament.

Ligament	Variable	Morphometrics
ACL	morphology	healthy 214 (51.6%) pathologic 158 (38.1%) * completely ruptured 43 (10.4%)
	anteroposterior diameter (mm)	9.94 ± 4.15 (2.22–22.65)
	mediolateral diameter (mm)	5.73 ± 2.20 (1.51–18.72)
	cross-sectional area (mm^2^)	43.68 ± 27.14 (4.91–245.20)
PCL	anteroposterior diameter (mm)	6.47 ± 1.58 (3.71–13.73)
	mediolateral diameter (mm)	11.48 ± 2.07 (5.63–19.10)
	cross-sectional area (mm^2^)	57.98 ± 16.42 (21.19–131.60)
	internal angle (°)	109.73 ± 12.71 (34.88–140.71)
MFL	one present 243 (58.6%)	aMFL 129 (31.1%) pMFL 114 (27.5%)
	both present 52 (12.5%)	
	completely missing 128 (28.9%)	
aMFL	missing 234 (56.4%) present 181 (43.6%)	
	length (mm)	31.96 ± 3.71 (22.77–42.25)
	anteroposterior width (mm)	2.83 ± 0.94 (0.99–6.13)
	cross-sectional area (mm^2^)	7.62 ± 4.73 (1.76–18.29)
pMFL	missing 249 (60.0%) present 166 (40.0%)	
	length (mm)	32.67 ± 4.44 (22.70–44.97)
	anteroposterior width (mm)	2.92 ± 1.03 (1.05–8.48)
	cross-sectional area (mm^2^)	7.70 ± 4.08 (1.56–20.90)
	angle (°)	31.27 ± 5.27 (17.67–48.04)

* partial tear 12 (2.9%), edematous swelling 44 (10.6%), cyst 11 (2.7%), mucoid hypertrophy 91 (21.9%).

## Data Availability

The raw data supporting the conclusions of this article will be made available by the authors on request.
